# Focus group-supported development and psychometric exploration of an instrument to assess perceived physical exertion in nursing students

**DOI:** 10.1186/s12912-024-02639-9

**Published:** 2024-12-30

**Authors:** Eva Lorenz, Eva Grüne, Johanna Popp, Klaus Pfeifer, Johannes Carl

**Affiliations:** 1 Department of Sport Science and Sport, Friedrich-Alexander-Universität Erlangen-Nürnberg, Erlangen, Germany; 2 Institute for Physical Activity and Nutrition, Deakin University, Geelong, Australia

**Keywords:** Questionnaire development, Exploratory factor analysis, Perceived physical exertion, Internal consistency, Cronbach’s alpha, Content validity

## Abstract

**Background:**

The physical demands of nurses during their work and education are high. In addition, shortage in nursing staff increases the individual workload. However, an appropriate tool to measure perceived physical exertion in nursing students is missing. Therefore, the goal of the present study was to design a questionnaire to assess perceived physical exertion in nursing students during their vocational education and to explore its factor structure.

**Methods:**

We initially conducted two parallel semi-structured focus group interviews with purposefully sampled nursing students to gain insights into their daily physical load. We coded the material and derived an initial set of 35 items (work-related and school-related). Subsequently, we conducted another semi-structured focus group interview with purposefully sampled nursing students of another school to cross-validate the items and refine the preliminary questionnaire according to their responses. To check the psychometric quality and factor structure of the questionnaire, we used data of 237 nursing students from 16 different nursing schools. We analyzed the items (*n* = 30) descriptively (including internal consistency via Cronbach’s α) and examined the structure of the questionnaire using exploratory factor analyses.

**Results:**

In the focus group interviews, we found different categories that play a role for perceived physical exertion in nursing vocational education: ‘general conditions’, ‘patient’, ‘additional load’, ‘locomotion’, ‘awkward postures’, ‘one-sided load’ and ‘others’. The factor analyses suggested three factors explaining the structure of the questionnaire. We registered satisfactory item statistics and good internal consistencies for all three factors: ‘relocating and handling of objects’ (α = 0.967); ‘personal care of patients’ (α = 0.910); ‘relocating patients’ (α = .809). The final questionnaire comprises 30 items (28 work-related, two school-related).

**Conclusion:**

The developed questionnaire provided initial evidence for content validity and internal consistency of the factors. The instrument can be used to detect perceived physical exertion in nursing students and thus help specifically address health-related problems. There is a need for a further confirmatory approach to cross-validate the questionnaire.

**Trail registration:**

Clinical trials NCT05817396.

**Supplementary Information:**

The online version contains supplementary material available at 10.1186/s12912-024-02639-9.

## Background

Demographic change challenges nurses’ working life and vocational education since it gradually causes an age shift towards an older society. In the medium and long term, these developments will substantially affect the quality provision of care [1]. To provide an adequate care to all the people and relieve the working staff, more future nurses are needed. The predominant shortage of nursing staff worldwide, including Germany, might add to challenging the provision of care [2, 3].

Accordingly, nursing staff is exposed to substantial levels of work demands with high physical loads and mental stress [2, 4–6]. This can manifest in physical complaints, such as musculoskeletal pain [7, 8]. Further, studies report on nurses’ health risk behavior. Lehmann et al. [9] observed in 2013 that nursing students have become more overweight and their alcohol consumption increased when compared to 2008. Chin et al. [7] found in their 2016 study from California that 48.7% of the participating nurses were either overweight or obese. However, the work environment can also contribute to demands posed to nurses. Working with sick persons is inherent to nursing, which includes nurses being exposed to pathogens and wounds [10, 11]. Nurses in surgery might even endure more health hazards, as this environment might expose them to radiation and toxic fluids [10]. Broetje et al. [12] identified key work demands in nursing. One of them is work overload. Another one includes the consequences of shift work, which is often part of the job profile, especially in clinics.

Several studies assessed psychological health in nurses and healthcare professionals, especially since the COVID-19 outbreak [13–15]. However, occupational physical demands could also contribute to decreasing health [16]. General reports on physically demanding activities are prominent in nursing and nursing students work. Those include tasks, such as standing, carrying and holding loads, as well as taking and remaining in awkward postures to meet their work demands [5, 11, 17]. Compared to other professions, those demands occur more frequently in care professions [5]. The contexts in which demand occurs are primarily patient related, but also include non-patient-related tasks [17]. However, there is little accurate data, especially regarding the *subjectively perceived physical exertion* in nursing students. This information is essential to improve endeavors for promoting physical activity in nursing students and thus develop more efficient interventions for strengthening health resources in this target group. Carl et al. [18] concluded that there might be a need to improve various competencies rather than to merely increase the physical activity volume of nursing students. However, this ambition requires more information on which situations cause *perceived physical exertion* at the workplace.

In line with the job demands-resource model [19, 20] we recognize physical demands as being possibly harmful for nursing workers’ health. Therefore, resources should be built to minimize *subjectively perceived physical exertion* caused by *physical load* during work. We understand *physical load* [17, 21] as the accumulation of physical stimuli that are placed externally on individuals, which results internally in *perceived physical exertion*. Our understanding of *subjectively perceived physical exertion* follows the definition of Borg: “Perceived exertion is the feeling of how heavy and strenuous a physical task is” [22, p. 8]. While *physical load* as an objectifiable variable can remain invariant across individuals, *perceived physical exertion* reflecting individuals internal processing of these stimuli differs according to factors like individual prerequisites or environmental factors [22, 23].

Assessing perceived physical exertion can help to understand the consequences of the demands, depending on the individual evaluation of the situation for the individuals health [24]. Several questionnaires partly assess perceived physical exertion but have too few questions regarding the construct, are not specifically targeting nursing students, or are conceptualized for persons with health conditions [25–28]. To date, there is, to the best of our knowledge, no instrument available that measures *perceived physical exertion* caused by physical loads during vocational education of nursing students. Consequently, we addressed this research gap for better understanding perceived physical exertion of nursing students. The development of a new instrument could contribute to identifying tasks perceived as physically exerting in nursing students. With this information, actions could be taken to counteract physical demands in vocational education and consequently lower perceived physical exertion in nursing students. Therefore, the goal of the present study was to develop such an instrument using a participatory approach and to explore the factor structure and internal consistency.

## Methods

### Study design

In the following, we present the procedure of the questionnaire development with a sequential exploratory mixed-methods design, to first explore perceived physically exerting activities of nursing students to develop and adapt an initial item-set using qualitative data and subsequently using quantitative data to explore factor structure and internal consistency of the instrument [29], supported by nursing students from Bavarian nursing schools, as members of the target group. Bavaria is one of 16 federal states in Germany, where it belongs to one of the most educated and largest regions [30, 31]. In 2020, the structure of nursing education was changed, whereby programs with specific focus on either pediatric, geriatric or patient care were combined within one education program with a broader focus (Act of the nursing professions, Ger: Pflegeberufereformgesetz, PflBRefG) [32]. In Germany, vocational education consists of two parts, a rather theoretical part in school and a more practical one in workplaces [33].

In summary, the development and exploration of the questionnaire involved the following steps: We started with (a) a literature search to identify possible demanding activities in work in general and in nursing. Based on the results, we (b) developed a focus-group interview guide and (c) held two focus groups in one nursing school on July 19th, 2022. In these focus groups, we asked the nursing students about activities causing perceived physical exertion within their vocational education. The recorded focus group interviews were then (d) transcribed and coded. Based on the results, we (e) developed a preliminary version of the questionnaire and (f) validated and adjusted it with the help of students from a second nursing school in a third focus group that took place on November 28th, 2022. The questionnaire was then (g) applied as part of the baseline survey in the TakeCare! project, which took place from April 16th until June 18th, 2023. (h) The data was used for an exploratory item analysis, (i) an exploratory factor analysis, (j) and the examination of the internal consistency (Cronbach’s α) of the factors.

The data in the present study stems from participants of the Physical Activity-related health Competence in Apprenticeship & Vocational Education (PArC-AVE) project [34, 35] and its follow-up TakeCare! project [36]. Both projects addressed the implementation of actions for physical activity promotion within nursing vocational education. Written informed consent was obtained by all participants in both projects.

The ethics committee of the Friedrich-Alexander-University Erlangen-Nürnberg approved the study PArC-AVE (January 15th, 2019; sign: 467_18 B) as well as the study TakeCare! (December 22nd, 2022; sign. 22-429-S). The Bavarian State Ministry for Education and Cultural Affairs approved the study TakeCare! (March 15th, 2023; sign. IV. 7-BO9106/144/9).

### Questionnaire development

With the help of three focus group interviews, we developed and validated the questionnaire. The participants of all focus groups were part of the PArC-AVE project. (a) The interview guide for the first two focus groups was based on a literature search. For this purpose, we searched SCOPUS, Web of Science and PubMed for articles on physical load at work in general as well as nursing specific workload. These demanding activities were collected and counted. (b) Based on this information, a guide for the first two focus groups was developed, which contained activities that were mentioned in several articles and found to be appropriate in the nursing context. The focus group interview guide included general questions about physical exertion the nursing students perceived during their vocational education (e.g., “Please think about your entire nursing vocational education. Which activities are particularly physically demanding for you?”), questions asking about the work with patients [10, 37, 38], work with additional load [5, 10, 17, 26, 27, 39–46], awkward postures [5, 11, 25–27, 40–47], locomotion [25, 41, 43], and finally questions about the physical exertion they perceive during theoretical sessions in the classroom.

We conducted (c) the two initial focus groups at one conveniently sampled Bavarian nursing school, where participants were purposefully sampled in order to have representatives of different primary employers and of the different years of the vocational education. EG and EL held the audiotaped focus group interviews, with the participants grouped around a table as recommended by Wong [48]. Alternatingly, one interviewed and the other one did the protocol. (d) EL and one student assistant transcribed the focus group interviews separately with MAXQDA (VERBI – Software. Consult. Sozialforschung. GmbH, Berlin, Germany) and double-checked the transcription of the other, respectively. We then coded the transcriptions according to qualitative content analysis by Kuckartz and Rädicker [49]. We formed the main categories as a combination of a deductive procedure with help of the focus group interview guide and an inductive procedure with the content of the interviews. Subsequently, we coded the focus group interview material according to the main categories. Afterwards, EL initially formed sub-categories inductively, based on the transcribed interviews and coded the material according to the sub-categories. After this, EG and JP double-checked both one half of the category assignment (four-eye principle). Together we discussed and revised the sub-category assignment until we reached consensus. (e) Based on these results and within team meetings, we developed the preliminary questionnaire with 35 items: 32 concerned work scenarios and three concerned school scenarios. The items were formed based on the focus group interviews, considering the frequencies of the coded text passages in the sub-categories. We formulated the items according to their content. The coded text passages showed that demanding activities were often linked to further circumstances, such as missing auxiliary tools or patient related restrictions. Therefore, we combined some item descriptions with those further circumstances to align the items more closely with practice. We tried to adhere to the technical language of the students as closely as possible.

The content of the preliminary questionnaire was (f) re-assessed with a semi-structured interview with an additional focus group of purposefully sampled students from another conveniently sampled nursing school in Bavaria. At the beginning, the participants of the focus group filled out the preliminary questionnaire. First, they rated the intensities of perceived physical exertion of the scenarios on a five-point Likert-scale ranging from not at all to very much (0–4). Second, they were asked to indicate the frequency of the respective scenario during their vocational education on a four-point Likert-scale (0–3). Third, the comprehensibility of the questions was enquired with dichotomous questions (yes or no). Afterwards, we performed the audio-taped focus group interview. The guide comprised questions about the comprehensibility of the items, the correspondence with their reality and suggestions for improvement, including a subjective estimation on whether items were unnecessary or missing. Likewise, two researchers conducted the focus group, with EL leading the focus group interview and JP taking protocols. Subsequently, the focus group interview was transcribed by a student assistant and double-checked by EL. The average scores of the intensity and the average frequency in nursing practice were multiplied to a final score. We excluded the items with a final score below two (score range after multiplication 0–12) and adjusted the items according to the comments of the third focus group.

### Psychometric exploration

The data collection was (g) embedded in the baseline survey of the Take Care! Project. Here, 16 nursing schools in Bavaria were recruited. As specified in the study protocol, the most important inclusion criteria of the schools were: (a) private or local sponsor, (b) having regularly between 33 and 64 students per year and (c) timely response by the school’s principal verifying participation with informed written consent [36]. Students of those schools were only included if they gave their informed written consent to participate. Of the 16 participating schools, three decided to provide their students a paper-pencil version of the questionnaire, where the others chose the online version via SoSci Survey (SoSci Survey GmbH, Munich, Germany). The recommendations for the sample size vary between 200 and 300 [50–52]. This is why we aimed for a sample size of about 300. With including students of 16 nursing schools in Bavaria, we assume to have included a wide range of different participants.

**Table 1:** Demographic characteristics of the survey participants

Variable	Description
Sample size	*n* = 237
Age	25.93 (± 9.78) years [range 16–54]
Gender	Female (76.8%), Male (21.1%), Diverse (1.3%) Missing (0.8%)
Body Mass Index	25.58 (± 6.03)
Highest school degree	Haupt-/Mittelschule (Middle School) (19.4%) Realschule/ Mittlere Reife (Secondary School) (47.3%) Abitur/Fachhochschulreife (University Entrance Qualification) (25.7%) Other (7.2%) Missing (0.4%)

Table 1 shows the demographic characteristics of the survey participants. Missing data were replaced by imputed values from an expectation maximization algorithm (assuming missing values to be missing at random after MCAR-test by Little) also including auxiliary variables (e.g., sociodemographic variables or variables correlating significantly with the present questionnaire) if the participant filled out at least 20% (6 items) of the questionnaire. We excluded participants who did not fill out at least 20% of the items.

After the (h) exploratory item analysis, we (i) conducted exploratory factor analysis (EFA). Since all factor-retention criteria have their strengths and weaknesses, several methods should be used and compared to determine the number of factors [53]. Therefore, we decided to use multiple methods, as the overall picture gives the best indication of the most likely number of factors. For statistical analysis, we mainly used SPSS, Version 29 (IBM Deutschland GmbH, Ehningen, Germany). We used R (R Foundation, Vienna, Austria) for the Empirical Kaiser Criterion (EKC) with the package semTools, version 0.5-6 [54], multivariate normality, with the package *mvn*, Version 5.9 [55], and Maximum Likelihood tests. We considered using the Maximum Likelihood Method (ML-Method) for EFA, but multivariate normal distribution in our data was violated in all tests (Mardia, Henze-Zirkel, Royston, Doornik-Hansen, and Energy). Accordingly, we decided to use Principal Axis Factoring (PAF) [56, 57]. We chose the oblique Promax rotation because it stands more strongly in line with correlated factors and, thus, cross-loadings reflecting combinations of complex movements relevant for mastering daily nursing tasks. We considered those as appropriate, since nursing work consists of different tasks combining complex movements [5, 17].

We used the syntaxes by O’Connor [58] for the Velicier’s Minimum-Average-Partial (MAP) test and the parallel analysis to determine the number of factors to extract, which provide information about a possible structure of the questionnaire. Additionally, we used the EKC Test, ML-Test, Scree-Test and Eigenvalue. We extracted the recommended number of factors for each procedure, compared them and then prioritized noticeable overlaps. Additionally, we compared those results constantly with content-related patterns and the interpretability of the factors [52]. (j) Finally, we calculated the Cronbach’s α values for the different factors. When factor naming was difficult, the items that loaded higher on a factor found more consideration. In naming the factors, we have oriented on the definitions of Durosaiye et al. [59].

## Results

### Development of the questionnaire and content validity

Each of the first two focus groups consisted of six nursing students from the same nursing school, with the first group including four females (67%) and the second group five females (83%). Both focus group interviews took about 75 min. The nursing students had different primary employers, for example: a clinic, an ambulatory service, a psychiatry, or a nursing home.

**Table 2:** Identified categories of the first two focus groups

Category (number of subcategories)	Number of coded text passages (approx. %)
General conditions (17)	110 (31.79%)
Patient (11)	96 (27.75%)
Additional load (5)	44 (12.72%)
Locomotion (3)	34 (9.83%)
Forced positions (6)	31 (8.96%)
One-sided load (5)	20 (5.78%)
Other (3)	11 (3.18%)
Total	346 (100%)

Note: The original designations of the categories were discussed in German; EL translated the categories for this article

Table 2 shows the main categories identified through the focus group interviews. Since the ‘general conditions’ appeared to influence the students considerably during their work, we decided to also code them, as the focus group interview material shows that these are inseparably interrelated with perceived physical exertion. These include, among others, attributes of patient-related (e.g., obesity and immobility), spatial (e.g., narrow rooms), and circumstantial (e.g., missing auxiliary tools and facility-related circumstances) influencing factors, totaling 31.79% of the coded text passages. The second largest category is ’patient’. This category often arose in the context of patient-related restrictions (e.g., patient is not in a position to help). These were often mentioned in combination with ‘awkward postures’ and ‘one-sided load’. The third highest category in count is ‘additional load’ that also occurred in combination with ‘locomotion’ in the focus group interviews, for example, while carrying objects. In the last category, ‘others’, we gathered further important activities, which we were not able to connect to one of the categories mentioned above. In general, nursing students mentioned that even though auxiliary tools are sometimes available they often are not used because of shortage of staff and time pressure. Text passages assigned to ‘additional load’ only referred to objects, patient-related weight like their body weight or weight of single extremities were assigned to ‘Patient’. Based on this categorization, we developed the first raw version of the questionnaire comprising 35 items, with 32 relating to perceived physical exertion at work and three relating to physical exertion at school. The items were formulated according to the content of the most frequently mentioned sub-categories identified in the focus group interviews. To maximize fit with practice, links to other categories, as stated above were integrated if considered necessary.

We revised this preliminary questionnaire with the help of a third focus group interview that was held at another nursing school, with a duration of 39 min. Six nursing students (33% female) participated. All of them had a clinic as a primary employer, but had to spend certain periods of their vocational education in other work environments (i.e., nursing homes and ambulatory service). After transcription and evaluation of the focus group interview and questionnaire, we reduced the questionnaire by five items due to the low relevance of the items according to the participants of the focus group, as well as the intensity x relevance calculation, as can be seen in an additional file [see Additional file 1]. Other items were adjusted in wording, for more precise formulation. Some adjustments were related to the time dimension and frequency to increase comprehensibility. There were no comments on the response options; therefore, we retained the 5-point Likert scale. If items were considered missing and this related to the first two focus groups, items were adjusted to also cover the missing tasks. This resulted in a questionnaire comprising 30 items: 28 of them related to work scenarios and two to school scenarios, as outlined in Table 5.

### Factor structure and internal consistency of the questionnaire

This newly developed questionnaire was used in the project TakeCare! for the first time. All of the following analyses were conducted with the data gathered at the first measurement time point (T0) of the project. The initial sample size of the participants was *n* = 267. Of those, 30 did not fill out at least the required 20% of the questionnaire and were, therefore, excluded. This resulted in a final participant number of *n* = 237. Table 3 shows the descriptive statistics of the data. The German version [see Additional file 2] and the English translation [see Additional file 3] of the questionnaire are displayed in the appendices. The mean values and item difficulties show that the values are grouped around the middle. The higher the item difficulty, the higher is the physical exertion the students perceive in the presented scenario.

**Table 3:** Descriptive statistics of the sample

Item nr.	Mean (0–4)	Item difficulty (0-100)	Standard deviation	Skewness	Kurtosis
1	2.26	56.5	1.173	− 0.298	− 0.757
2	2.74	68.5	1.377	− 0.724	− 0.780
3	2.40	60.0	1.203	− 0.403	− 0.665
4	1.75	43.8	1.262	0.327	− 0.867
5	2.29	57.3	1.094	− 0.216	− 0.527
6	2.07	51.8	1.231	− 0.057	− 0.959
7	1.78	44.5	1.290	0.181	-1.002
8	2.27	56.8	1.282	− 0.194	-1.011
9	1.79	44.8	1.224	0.205	− 0.931
10	1.29	32.3	1.284	0.600	− 0.728
11	1.88	47.0	1.339	0.269	-1.107
12	2.01	50.3	1.116	− 0.006	− 0.601
13	2.10	52.5	1.117	0.020	− 0.653
14	1.47	36.8	1.365	0.520	− 0.944
15	1.37	34.3	1.331	0.582	− 0.887
16	1.47	36.8	1.338	0.463	− 0.968
17	1.66	41.5	1.391	0.306	-1.132
18	1.27	31.8	1.413	0.738	− 0.839
19	1.87	46.8	1.237	0.028	− 0.989
20	1.77	44.3	1.303	0.230	-1.027
21	1.33	33.3	1.390	0.730	− 0.766
22	1.52	38.0	1.355	0.500	− 0.931
23	1.40	35.0	1.382	0.646	− 0.831
24	1.31	32.8	1.488	0.795	− 0.846
25	1.34	33.5	1.433	0.734	− 0.841
26	1.56	39.0	1.366	0.507	− 0.949
27	1.50	37.5	1.347	0.498	− 0.929
28	1.67	41.8	1.309	0.289	-1.013
02_01	2.14	53.5	1.357	− 0.186	-1.153
02_02	1.71	42.8	1.292	0.289	− 0.919

Note: *N* = 237, standard error in skewness for each item 0.158, standard error in kurtosis in each item 0.315

### Exploratory factor analysis

**Table 4:** Results of the methods to determine the number of extracted factors

Method	Number of extracted factors
Eigenvalue	5
Scree test	2
Velicier’s MAP test 1976/2000	3/4
Parallel analysis PCA/PAA	2/4
ML test	15
EKC	13

Note: MAP = Minimum-Average-Partial; PCA = Principal Component Analysis; PAA = Principal Axis Analysis/Common Factor Analysis; ML = Maximum Likelihood; EKC = Empirical Kaiser Criterion

Table 4 shows the numbers of factors for extraction from the different methods. The results were heterogeneous, suggesting to either extract two or four factors. However, in conjunction with various theoretical considerations, three factors were most suitable. The considerations were as follows: The number of factors that should be extracted showed an ambivalent picture. Since Eigenvalue and scree-test are sometimes criticized as outdated methods [52], we decided to more strongly prioritize the remaining procedures. The results of the ML-Method suggested 15 factors and the EKC 13 factors. Since the questionnaire comprised 30 items, the results of the ML-Method and EKC were not considered, because this would have reduced the factors of only about half and, therefore, conflicted with the goal to extract factors substantially reducing information. Velicier’s MAP test (2000) and Parallel analysis PAA indicated that four factors might be a reasonable number, but this four-factor solution displayed a Heywood case [60]. Overextraction of factors might be one cause for a Heywood case [61]. Therefore, we tested a three-factor solution that not only avoided this problem but also assigned more than four items to each factor. It is regarded as desirable to have a sufficient amount of items assigned to a factor, which was not met in the four-factor solution [52, 62]. Both the three-factor and the four-factor solutions showed high configural overlap. The slight difference between both variations is that two factors with few items were merged into one and single items were also added to this factor that still loaded higher than 0.30 on their original factor and were therefore displayed as cross-loading items. Importantly, the core of the configurations, especially between factors ‘relocating and handling of objects’ and ‘relocating patients’, was comparably stable across the four and three-factor solutions. We finally decided to favor the three-factor solution, as the discussed empirical endorsement was substantiated by theoretical plausible interpretability. As the plausible interpretability appeared to apply to the three-factor solutions, we used it for our EFA. The exact results of the tests can be found in an additional file [see Additional file 4].

The Kaiser-Meyer-Olkin (KMO) value was 0.935 and the significance of the Bartlett test was *p* < .001, both indicating good suitability of the data for factor analysis. The measure of sampling adequacy (MSA) coefficient for each item was sufficient (0.598 − 0.974).

**Table 5:** Results of the exploratory factor analysis

Sample matrix^a^					
			Factor		
Item no.	Items		1: Relocating and handling of objects	2: Personal care of patients	3: Relocating patients
25	You are lifting up objects up to 5 kg (e.g., carton with medication).		**0.990**	-0.089	-0.012
22	You are transporting objects above 5 kg (e.g., luggage of a patient) from one ward to the next.		**0.971**	-0.094	0.031
26	You are lifting up objects above 5 kg (e.g., rinsing solutions, oxygen bottles).		**0.945**	-0.064	0.045
24	You are pushing a patient in a wheelchair from one ward to the next.		**0.911**	0.009	-0.086
21	You are transporting objects up to 5 kg (e.g., tea, towels, food trays) across the ward.		**0.894**	0.020	0.017
27	You are holding objects up to 5 kg (e.g., infusion bags) over 1 min.		**0.890**	-0.121	0.101
28	You are holding objects above 5 kg (e.g., rinsing solutions) over 1 min.		**0.881**	-0.210	0.179
23	You are pushing an empty bed across the ward.		**0.828**	0.100	-0.077
02_02	You are carrying school things (e.g., bag, folder, books/tablet) with you on a usual school day.		**0.693**	0.047	-0.039
18	You are disinfecting the surfaces in a room (e.g., television, nurse call button) within 2 min.		**0.640**	0.260	-0.071
20	You are climbing the stairs over 2 floors.		**0.561**	0.230	-0.106
17	You are putting clean sheets on an empty bed within 5 min.		**0.536**	0.266	-0.079
4	You are moving a partially mobile patient (approx. 80 kg) from bed to a wheelchair with their help.		**0.478**	0.214	**0.406**
14	You are measuring the blood pressure of 20 patients one after another.		**0.465**	**0.411**	-0.147
02_01	You are sitting in class on a usual school day.		**0.407**	0.053	-0.078
12	You are cleaning the wound of a patient (approx. 80 kg) in an area that is difficult to reach (e.g., calf, buttocks).		-0.086	**0.787**	0.171
13	You are changing the bandage on a leg of a patient (approx. 80 kg) while holding up the leg.		-0.124	**0.626**	0.138
9	You are assisting a partially mobile patient (approx. 80 kg) in body care in a narrow bathroom.		0.142	**0.559**	0.231
10	You are emptying indwelling catheters of 8 patients one after another.		**0.414**	**0.488**	-0.075
15	You are connecting and disconnecting infusions of 20 patients one after another.		**0.359**	**0.472**	-0.110
16	You are preparing the medication for 20 patients.		**0.389**	**0.438**	-0.060
11	You are putting on compression stockings on a patient (approx. 80 kg) in bed.		**0.420**	**0.426**	0.031
7	You are washing an immobile patient (approx. 80 kg) in bed without their help.		0.242	**0.403**	**0.342**
19	You are standing in one place for 20 min (e.g., during surgery, while documentation).		0.257	**0.386**	-0.096
1	You are positioning an immobile patient (approx. 80 kg) in bed without their help.		0.272	-0.110	**0.831**
3	You are moving an immobile patient (approx. 80 kg) from bed to a wheelchair without their help.		0.029	0.059	**0.737**
2	You are positioning an immobile patient with obesity (approx. 120 kg) in bed without their help		**-0.469**	-0.066	**0.656**
5	You are moving a partially mobile patient with obesity (approx. 120 kg) from bed to a wheelchair with their help.		-0.136	0.106	**0.597**
8	You are washing an immobile patient with obesity (approx. 120 kg) in bed without their help.		-0.243	0.232	**0.442**
6	You are moving a partially mobile patient (approx. 80 kg) from the ground to bed together with a second nurse after a fall.		0.189	**0.327**	**0.328**

Note: Extraction method: Principal Axis Factoring, Rotation method: Promax with Kaiser-Normalization

a. The rotation is converged in 6 iterations

Table 5 shows the results of the factor analysis. The results are sorted according to the height of the factor loadings. Those higher than 0.30 are written in bold text. Most of the items loaded high on the first factor. Item two was the only item with an unclear factor assignment with medium negative load on the first factor and a medium positive load on the second. The factor correlation matrix is displayed in an additional file [see Additional file 5].

### Internal consistency

The internal consistency was calculated for the sub-scales. Factor one ‘relocating and handling of objects’ comprises 19 items, with a high Cronbach’s α value (α = 0.967). The second factor ‘personal care of patients’ includes eleven items and shows good internal consistency (α = .910). The internal consistency of factor three ‘relocating patients’, which includes eight items, is also high (α = .809). The final German version of the questionnaire can be found in Additional file 2 and for illustration purposes also an English language translation in Additional file 3.

## Discussion

The aim of this study was to develop a new questionnaire assessing perceived physical exertion in nursing students, and to examine the factor structure via exploratory factor analyses, the content validity, and internal consistency of this questionnaire. As part of the questionnaire development, several topics arose within the first two focus group interviews that helped gather information about the physical load that nursing students face during their vocational education. In summary, the focus group interviews revealed that nursing students have to perform complex movements during their work, which are difficult to clearly assign to a single category. Therefore, we decided to formulate the items by combining the forms of movement as they were mentioned in the focus group interviews. This assured that the movements were not separated into their single components, but rather captured the complex movements that occur in vocational education of nurses. Several topics are comparable to those arising in literature. For example, ‘positioning of patients’ and ‘personal care’ among others were also found as demanding activities [59]. At school, long sitting was a problem that was mentioned by nursing students, alongside prolonged standing in one spot. This relates also to literature in which long sitting periods and long static activities were mentioned as challenging tasks [25, 26, 42, 44, 45]. The nursing students also stated that, due to time pressure and shortage of staff, they did many tasks without auxiliary tools that are usually provided by employers. In these stressful situations, their tasks at work are often not save and might affect their health [63].

Twelve nursing students participated in the first two focus groups that served as a basis for the development of the questionnaire. We decided to use focus group interviews for the questionnaire development, because with this method ideas can be generated utilizing a discussion format, where the technical language used by the target group becomes clear [48, 64]. With this information, the items can be formulated using the technical language of the target group and therefore reducing misunderstanding of items. With the purpose of adapting the questionnaire, a third focus group was conducted, which consisted of six additional nursing students. Altogether, 18 nursing students participated in the focus groups that served for the development of the questionnaire. We assume this number to be sufficient to counteract the subjective view of single persons.

Furthermore, content validity through the lens of nursing students can be assumed, because we recruited three focus groups of experts in the field [65], namely nursing students during their vocational education. With the help of the first two groups, we developed the questionnaire [66]. For content validation, Almanasreh et al. [66] recommended to use between five to ten experts. In our case, six nursing students participated. We considered further principles of item development, such as the definition of a clear time frame, the avoidance of double negation, or the inclusion of suggestive questions [52].

For the statistical analysis of our study that included exploratory item and exploratory factor analyses, the sample of nursing students participating in the survey included 76.8% females, which is very close to the global general gender distribution of nurses with 76.9% being women [67]. Also, in our study, the participating nurses showed an elevated BMI with a mean of 25.6, which is also common in this profession [68]. Therefore, our sample appeared to adequately represent the target population. In general, a negative kurtosis shows a rather flatter distribution than normal distribution [69]. Consequently, all our items showed a flatter distribution in exploratory item analysis. Combined, the descriptive statistics showed no outliers, indicating that the items are formulated neither too easy nor too difficult.

As our study shows, EFA might bring challenges in interpretability. However, after careful consideration of the factor retention criteria’s results with plausible interpretability, we found that the questionnaire comprises three factors. Those are ‘relocating and handling of objects’, ‘personal care of patients’, and ‘relocating patients’. Factor names relate to the definitions of Durosaiye et al. [59]. For instance, they include in personal care: “Washing and ensuring patients are clean, dressed, and well, including toileting and catheterization” [59, p. 280]. The three identified factors subsume different tasks occurring in nursing apprenticeship. ‘Relocating and handling of objects’ encompasses carrying, pushing, lifting, holding, transporting and more complex movements, where ‘relocating patients’ encompasses complex movements of positioning and moving patients. The dimension ‘personal care of patients’ emphasizes physical exertion in interactive social constellations. Which specific movements (lifting, pushing, holding forced positions) those tasks include can be theoretically assumed but would require empirical testing, utilizing methods to determine criterion validity.

Due to the fact that removing items might affect validity [70], item two was not removed from the questionnaire. Even though it had an ambiguous factor assignment to factor one with a medium negative and factor three with a medium positive loading. Additionally, the nursing students in the third focus group did not exclude the item, thus encouraging us to maintain it. In future considerations, however, it should be checked whether item two fits into the results. If that is not the case, considerations should be made whether the item is better removed.

The internal consistency of the questionnaire is high, with Cronbach’s α values ranging from 0.809 to 0.967, which indicates that variables in the grouped constellations can be measured in a sufficiently reliable manner. Furthermore, some of the secondary quality criteria are also fulfilled by the questionnaire. The test can be considered economic because this measure is low in time and costs [71]. We consider this to be fulfilled, since our questionnaire only comprised 30 items and takes about 7–10 min for completion. However, we chose a questionnaire as an assessment tool due to the subjective character of the construct. This format also appeared to be more economic to gather larger amounts of data [72]. There exist several questionnaires assessing similar constructs, such as the physical workload questionnaire [25], which is designed to assess the range of physical demands during work, but not the exertion that the responders perceive during those demands and it is not specific to nursing. Another extensive measure that does a risk assignment for physical load in German language includes several tasks that occur in nursing, however it is not nursing specific [41]. The structured multidisciplinary work evaluation tool [45] measures physical workload in nursing assistants but does not ask about specific situations, rather about specific problems with movements occurring in physical work. We however, wanted to specify specific situations in order to better visualize the situations and give the nursing students an example they can recognize immediately. Since nursing students are the workforce of tomorrow, it is especially important to consider this target group, also in view of the increasing burden of demographic change. This is why we saw the need to develop a new questionnaire measuring perceived physical exertion in nursing students.

When considering the questionnaire in the context of the job-demands-resource model [24], the items represent the demands that are posed to the individuals and the answers allow to draw implications for individual’s respective resources. Those resources could be personal or organizational, including auxiliary tools or colleagues which can help facilitate a task. In the future, if the questionnaire is successfully undergoing further analyses, it can be used to generate knowledge about the specific fields in which nursing students in general or an individual nursing student might have benefits or difficulties regarding their perceived physical exertion. Accordingly, interventions can be designed or actions can be taken to combat negative consequences from excessive perceived physical exertion. For example, working groups could be formed in the workplace in which the various strengths and weaknesses of the members could be balanced out, thus reducing the overall perceived physical exertion for the group or individuals. On this basis, steps can be taken to further decrease the overall level of perceived physical exertion in nursing vocational education. Another option is to design interventions strengthening personal resources, such as competence-based interventions. If demanding activities can be identified, specific competencies should be promoted through the systems in which nurses work. Those can support individuals meeting physical demands in a competent manner, as for instance considered in the physical activity-related health competence model [73, 74]. In order to avoid job retention and enhance job adherence and attractiveness, solutions need to be found to reduce the burden of the job.

Future research should examine whether the physical exertion perceived during vocational education depends significantly on individuals’ resources to master vocational demands. Addressing this gap would not only deliver arguments for or against the relevance of specific coping mechanisms, such as getting a second person for help, but also provide further evidence regarding the criterion validity of the present instrument. As a further step, a confirmatory approach is indicated to re-validate the factor structure determined in our study. If the basic structure of this instrument proves to be valid in a confirmatory approach, future initiatives may lead to collecting data for norm values enabling comparisons with other data of the general nursing student population and over time. When using a confirmatory approach, it should be specifically considered whether item two is appropriate and should remain in the questionnaire.

There are several limitations in our study: First, we developed a questionnaire, which is a self-report measure. In such measure, recall bias and social desirability might affect the answers, which might influence the validity of the instrument. That is why there is a need for further validation. However, the purpose of this questionnaire is to measure perceived physical exertion. Therefore, we consider a questionnaire to be an appropriate tool, since the measured construct is a subjective one. Second, the questionnaire is formulated in German language. We tried to adhere closely to the technical language of the German nursing students to generate a comprehensible tool for assessing perceived physical exertion in this population. However, there is no validated English translation, which limits the operational capability of the questionnaire, as preliminary psychometric suggestions can only be given for the German speaking area so far. Third, we formulated items that represented specific situations, on the one hand, but allow a self-responsible selection on the mode how these challenges were mastered. This leaves room for individuals to incorporate their individual solutions into the answers. This might be, however, a source of error, when participants imagine different solution strategies. Nevertheless, we found it important to leave room for incorporating different solution strategies, to not predetermine the difficulty of the items.

## Conclusions

For the development of a new instrument to measure nursing students perceived physical exertion, we deliberately favored a participatory approach with representatives of the target group. This procedure aimed to foster content validity through the application of focus group interviews with the target population. We identified three factors within the questionnaire and consistently registered high reliability values (internal consistency via Cronbach’s α values). The first factor is ‘relocating and handling of objects’, the second comprises ‘personal care of patients’, and the third ‘relocating patients’. The questionnaire adds to the scientific field, by providing a validated and reliable tool for measuring perceived physical exertion in nursing students. As previously mentioned, nurses report poor health, high perceived physical exertion, and issues pertaining to the work environment. The present questionnaire can help to illuminate individual challenges with specific working situations among nursing students. To the best of our knowledge, this is the first questionnaire for this population that fills the scientific gap for such an evaluation tool. Future research should cross-validate the questionnaire in another nursing population using confirmatory factor analysis and check for re-test reliability to underpin recommendations for its application in real-world settings.

## Electronic supplementary material

Below is the link to the electronic supplementary material.

Supplementary Material 1

Supplementary Material 2

Supplementary Material 3

Supplementary Material 4

Supplementary Material 5

## Data Availability

The primary data are available from the corresponding author upon request.

## Abbreviations

EFA: Exploratory Factor Analysis
EM: Expectation Maximization
MCAR: Missing Completely at random
MAR: Missing at random
EKC: Empirical Kaiser Criterion
MAP: Minimum-Average-Partial
PAF: Principal Axis Factoring
KMO: Kaiser-Meyer-Olkin
MSA: Measure of Sampling Adequacy
PCA: Principal Component Analysis
PAA: Principal Axis Analysis

## References

[1] Nowossadeck E. Pflegekräfte in Zeiten des Demografischen Wandels. Probleme, Herausforderungen und Lösungsstrategien. Bundesgesundheitsbl. 2013;56(8):1037–9. 10.1007/s00103-013-1741-2 23884517

[2] Tamata AT, Mohammadnezhad M. A systematic review study on the factors affecting shortage of nursing workforce in the hospitals. Nurs Open. 2023;10(3):1247–57. 36303066 10.1002/nop2.1434 PMC9912424

[3] Bundesagentur für Arbeit. Arbeitsmarktsituation im Pflegebereich. Nürnberg (DE). 2023:23.

[4] Cockerham M, Kang DH, Beier ME. Consecutive Shifts: A Repeated Measure Study to Evaluate Stress, Biomarkers, Social Support, and Fatigue in Medical/Surgical Nurses. Behav Sci (Basel). 2023;13(7):571. 37504018 10.3390/bs13070571 PMC10376272

[5] Lück M, Melzer M. Arbeitsbedingungen in der Alten- und Krankenpflege - Höhere Anforderungen, mehr gesundheitliche Beschwerden. BIBB/BAuA-Faktenblatt 31. Dortmund (DE): Bundesanstalt für Arbeitsschutz und Arbeitsmedizin (BAuA). 2020:2.

[6] Chesak SS, Cutshall SM, Bowe CL, Montanari KM, Bhagra A. Stress management interventions for nurses: critical literature review. J Holist Nurs. 2019;37(3):288–95. 31014156 10.1177/0898010119842693

[7] Chin DL, Nam S, Lee SJ. Occupational factors associated with obesity and leisure-time physical activity among nurses: a cross sectional study. Int J Nurs Stud. 2016;57:60–9. 27045565 10.1016/j.ijnurstu.2016.01.009 PMC4871118

[8] Hämmig O. Work- and stress-related musculoskeletal and sleep disorders among health professionals: a cross-sectional study in a hospital setting in Switzerland. BMC Musculoskelet Disord. 2020;21(1):319. 32438929 10.1186/s12891-020-03327-w PMC7243303

[9] Lehmann F, von Lindeman K, Klewer J, Kugler J. BMI, physical inactivity, cigarette and alcohol consumption in female nursing students: a 5-year comparison. BMC Med Educ. 2014;14:82. 24742064 10.1186/1472-6920-14-82 PMC3997793

[10] Berentzen J, Lennartz S. Arbeitsplatz Operationsabteilung: Physische Belastungen für OP-Personal – Möglichkeiten der Gesundheitsförderung und Prävention. Op-Journal. 2010;26(01):48–53.

[11] Drupp M, Meyer M. Belastungen und Arbeitsbedingungen bei Pflegeberufen – Arbeitsunfähigkeitsdaten und ihre Nutzung im Rahmen eines Betrieblichen Gesundheitsmanagements. In: Jacobs K, Kuhlmey A, Greß S, Klauber J, Schwinger A, editors. Pflege-Report 2019. Berlin, Heidelberg: Springer; 2020. pp. 23–47.

[12] Broetje S, Jenny GJ, Bauer GF. The Key Job Demands and Resources of Nursing Staff: an Integrative Review of Reviews. Front Psychol. 2020;11:84. 32082226 10.3389/fpsyg.2020.00084 PMC7005600

[13] Pavek KU, Steege LM, Kwekkeboom K. Testing Content Validity of Nursing Stress Scales: Do They Reflect Current Practice? SAGE Open. 2022;12(3).

[14] Shaukat N, Ali DM, Razzak J. Physical and mental health impacts of COVID-19 on healthcare workers: a scoping review. Int J Emerg Med. 2020;13(1):40. 32689925 10.1186/s12245-020-00299-5 PMC7370263

[15] Preti E, Di Mattei V, Perego G, Ferrari F, Mazzetti M, Taranto P, et al. The Psychological Impact of Epidemic and Pandemic Outbreaks on Healthcare Workers: Rapid Review of the Evidence. Curr Psychiatry Rep. 2020;22(8):43. 32651717 10.1007/s11920-020-01166-z PMC7350408

[16] Holtermann A, Krause N, van der Beek AJ, Straker L. The physical activity paradox: six reasons why occupational physical activity (OPA) does not confer the cardiovascular health benefits that leisure time physical activity does. Br J Sports Med. 2018;52(3):149–50. 28798040 10.1136/bjsports-2017-097965

[17] Alghamdi MG. Nursing workload: a concept analysis. J Nurs Manag. 2016;24(4):449–57. 26749124 10.1111/jonm.12354

[18] Carl J, Grüne E, Popp J, Pfeifer K. Physical Activity Promotion for Apprentices in Nursing Care and Automotive Mechatronics-Competence Counts More than Volume. Int J Environ Res Public Health. 2020;17(3). 10.3390/ijerph17030793 PMC7037564 32012835

[19] Demerouti E, Bakker AB, Nachreiner F, Schaufeli WB. The job demands-resources model of burnout. J Appl Psychol. 2001;86(3):499–512. 11419809

[20] Bakker AB, Demerouti E. Job demands-resources theory: Taking stock and looking forward. J Occup Health Psychol. 2017;22(3):273–85. 27732008 10.1037/ocp0000056

[21] Load. Overload, and recovery in the Athlete: Select Issues for the Team Physician-A Consensus Statement. Curr Sports Med Rep. 2019;18(4):141–8. 30969240 10.1249/JSR.0000000000000589

[22] Borg G. Borg’s perceived exertion and pain scale. United States of America: Human Kinetics; 1998.

[23] Andersen LL, Clausen T, Persson R, Holtermann A. Perceived physical exertion during healthcare work and risk of chronic pain in different body regions: prospective cohort study. Int Arch Occup Environ Health. 2013;86(6):681–7. 22878558 10.1007/s00420-012-0808-y

[24] Bakker AB, Demerouti E. The Job Demands-Resources model: state of the art. J Manage Psychol. 2007;22(3):309–28.

[25] Bot SD, Terwee CB, van der Windt DA, Feleus A, Bierma-Zeinstra SM, Knol DL, et al. Internal consistency and validity of a new physical workload questionnaire. Occup Environ Med. 2004;61(12):980–6. 15550603 10.1136/oem.2003.011213 PMC1740683

[26] Slesina W. FEBA: Fragebogen zur subjektiven Einschätzung der Belastung am Arbeitsplatz ASER Institut; 2009 www.rueckenkompass.de. Accessed 07 Nov 2023.

[27] Kjønø LG, Killingmo RM, Vigdal ØN, Grotle M, Storheim K. Assessing physical workload among people with musculoskeletal disorders: validity and reliability of the physical workload questionnaire. BMC Musculoskelet Disord. 2022;23(1):282. 35331205 10.1186/s12891-022-05222-y PMC8944019

[28] Onega LL. Helping Those Who Help Others: the Modified Caregiver Strain Index. AJN. 2008;108(9):62–9. 18756161 10.1097/01.NAJ.0000334528.90459.9a

[29] Creswell JW, Plano Clark VL. Designing and Conducting Mixed Methods Research 2010.

[30] INSM. Bildungsmonitor 2024 2024 https://insm.de/bildungsmonitor-2024. Accessed 07 Nov 2024.

[31] Statistisches Bundesamt. Verwaltungsgliederung am 31.03.2024 (1. Quartal) 2024 https://www.destatis.de/DE/Themen/Laender-Regionen/Regionales/Gemeindeverzeichnis/Administrativ/Archiv/Verwaltungsgliederung/Verwalt1QAktuell.html. Accessed 07 Nov 2024.

[32] Weiß T, Meißner T, Kempa S. Pflegeberufereformgesetz (PflBRefG) 2018.

[33] Grüne E, Popp J, Carl J, Pfeifer K. What do we know about physical activity interventions in vocational education and training? A systematic review. BMC Public Health. 2020;20(1):978. 32571295 10.1186/s12889-020-09093-7 PMC7309979

[34] Popp J, Carl J, Grüne E, Semrau J, Gelius P, Pfeifer K. Physical activity promotion in German vocational education: does capacity building work? Health Promot Int. 2020;35(6):1577–89. 32105312 10.1093/heapro/daaa014 PMC7785309

[35] Grüne E, Popp J, Carl J, Semrau J, Pfeifer K. Examining the sustainability and effectiveness of co-created physical activity interventions in vocational education and training: a multimethod evaluation. BMC Public Health. 2022;22(1):765. 35428289 10.1186/s12889-022-13133-9 PMC9011375

[36] Carl J, Grüne E, Popp J, Hartung V, Pfeifer K. Implementation and dissemination of physical activity-related health competence in vocational nursing training: study protocol for a cluster-randomized controlled intervention trial. Trials. 2024;25(1):322. 38750590 10.1186/s13063-024-08153-2 PMC11094863

[37] van Dam K, Meewis M, van der Heijden BI. Securing intensive care: towards a better understanding of intensive care nurses’ perceived work pressure and turnover intention. J Adv Nurs. 2013;69(1):31–40. 22420721 10.1111/j.1365-2648.2012.05981.x

[38] Miranda DR, Nap R, de Rijk A, Schaufeli W, Iapichino G. Tiss Working Group. Nursing activities score. Crit Care Med. 2003;31(2):374–82. 12576939 10.1097/01.CCM.0000045567.78801.CC

[39] Höhmann U, Lautenschläger M, Schwarz L. Belastungen im Pflegeberuf: Bedingungsfaktoren, Folgen und Desiderate. In: Jacobs K, Kuhlmey A, Greß S, Klauber J, Schwinger A, editors. Pflege-Report 2016. Stuttgart (DE). 2016. pp. 73–89.

[40] Breinbauer M. Arbeitsbedingungen und Arbeitsbelastungen in der Pflege [dissertation]. Mainz (DE): Gutenberg-Universität Mainz; 2020.

[41] Bundesanstalt für Arbeitsschutz und Arbeitsmedizin (BAuA). Gefährdungsbeurteilung bei physischer Belastung - die neuen Leitmerkmalmethoden (LMM) -Kurzfassung. Dortmund (DE); 2019 Oct. p.2.

[42] Lück M, Hünefeld L, Brenscheidt S, Bödefeld M, Hünefeld A. Grundauswertung der BIBB/BAuA Erwerbstätigenbefragung 2018 Vergleich zur Grundauswertung 2006 und 2012. Dortmund/Berlin/Dresden (DE); 2018 Dec. p.68.

[43] Hartmann B, Weber B, Ellegast R, Jäger M, Schick R, Spallek M. Die „Checkliste 2021" für physische Belastungen bei der Arbeit. Zbl Arbeitsmed. 2021;71(3):144–56.

[44] Zok K. Gesundheitliche Beschwerden und Belastungen am Arbeitsplatz Ergebnisse aus Beschäftigtenbefragungen. Berlin (DE): KomPart; 2010. p. 146.

[45] Haraldsson P, Areskoug-Josefsson K, Rolander B, Strengbom E, Jonker D. Comparing the Structured Multidisciplinary work Evaluation Tool (SMET) questionnaire with technical measurements of physical workload in certified nursing assistants in a medical ward setting. Appl Ergon. 2021;96:103493. 34116412 10.1016/j.apergo.2021.103493

[46] Luick RS. Körperliche Belastungen am Arbeitsplatz und ihre Folgen. In: Hahnzog S, editor. Betriebliche Gesundheitsförderung. Wiesbaden (DE): Springer Gabler; 2014. pp. 189–99.

[47] Yeung SS, Genaidy A, Deddens J, Sauter S. The relationship between protective and risk characteristics of acting and experienced workload, and musculoskeletal disorder cases among nurses. J Saf Res. 2005;36(1):85–95. 10.1016/j.jsr.2004.12.002 15752486

[48] Wong LP. Focus group discussion: a tool for health and medical research. Singap Med J. 2008;49(3):256–60. 18363011

[49] Kuckartz U, Rädicker S. Qualitative inhaltsanalyse. Methoden, Praxis, Computerunterstützung. Weinheim Basel (DE): Verlagsgruppe Beltz; 2022.

[50] Fabrigar LR, Wegener DT, MacCallum RC, Strahan EJ. Evaluating the use of exploratory factor analysis in psychological research. Psychol Methods. 1999;4(3):272–99.

[51] Field A. Discovering statistics using SPSS. SAGE; 2009.

[52] Bühner M. Einführung in die Test- und Fragebogenkonstruktion. München (DE): Pearson Education Deutschland GmbH; 2021.

[53] Brandt H. Exploratorische Faktorenanalyse (EFA). In: Moosbrugger H, Kelava A, editors. Testtheorie und Fragebogenkonstruktion. Berlin, Heidelberg: Springer; 2020. pp. 575–614.

[54] Jorgensen TD, Pornprasertmanit S, Schoemann AM, Rosseel Y, semTools. Useful tools for stuctural equation modeling. R package version 0.5-6 2022 https://CRAN.R-project.org/package=semTools. Accessed 14 Nov 2024.

[55] Korkmaz S. MVN: An R Package for Assessing Multivariate Normality 2021 https://cran.r-project.org/web/packages/MVN/vignettes/MVN.html. Accessed 05 Nov 2024.

[56] Goretzko D, Pham TTH, Bühner M. Exploratory factor analysis: Current use, methodological developments and recommendations for good practice. Curr Psychol. 2019;40(7):3510–21.

[57] Schreiber JB. Issues and recommendations for exploratory factor analysis and principal component analysis. Res Social Adm Pharm. 2021;17(5):1004–11. 33162380 10.1016/j.sapharm.2020.07.027

[58] O’Connor BP. SPSS and SAS programs for determining the number of components using parallel analysis and Velicer’s MAP test. Behav Res Methods Instrum Comput. 2000;32(3):396–402. 11029811 10.3758/bf03200807

[59] Durosaiye IO, Hadjri K, Liyanage CL, Bennett K. A matrix for the qualitative evaluation of nursing tasks. J Nurs Manag. 2018;26(3):274–87. 29164791 10.1111/jonm.12543

[60] Heywood HB. On finite sequences of real numbers. Proc R Soc Lond, ser A. Math Phys Eng Sci. 1931;134(824):486–501.

[61] Cooperman AW, Waller NG. Heywood you go away! Examining causes, effects, and treatments for Heywood cases in exploratory factor analysis. Psychol Methods. 2022;27(2):156–76. 34197140 10.1037/met0000384

[62] Watkins MW. Exploratory Factor Analysis: A Guide to Best Practice. JBP. 2018;44(3):219–46.

[63] Humphries N, Morgan K, Conry MC, McGowan Y, Montgomery A, McGee H. Quality of care and health professional burnout: narrative literature review. Int J Health Care Qual Assur. 2014;27(4):293–307. 25076604 10.1108/IJHCQA-08-2012-0087

[64] Nassar-McMillan S, Dianne Borders L. Use of Focus Groups in Survey Item Development. TQR. 2002;7(1):1–12.

[65] Himme A. Gütekriterien der Messung: Reliabilität, Validität und Generalisierbarkeit. In: Albers S, Klapper D, Konradt U, Walter A, Wolf J, editors. Methodik der empirischen Forschung. Wiesbaden (DE): Springer Gabler; 2007. pp. 375–90.

[66] Almanasreh E, Moles R, Chen TF. Evaluation of methods used for estimating content validity. Res Social Adm Pharm. 2019;15(2):214–21. 29606610 10.1016/j.sapharm.2018.03.066

[67] Kharazmi E, Bordbar N, Bordbar S. Distribution of nursing workforce in the world using Gini coefficient. BMC Nurs. 2023;22(1):151. 37147626 10.1186/s12912-023-01313-w PMC10161512

[68] Sadali UB, Kamal KKBN, Park J, Chew HSJ, Devi MK. The global prevalence of overweight and obesity among nurses: a systematic review and meta-analyses. J Clin Nurs. 2023;32(23–24):7934–55. 37775510 10.1111/jocn.16861

[69] Fahrmeir L, Heumann C, Künstler R, Pigeot I, Tutz G. Univariate Deskription und Exploration von Daten. In: Fahrmeir L, Heumann C, Künstler R, Pigeot I, Tutz G, editors. Statistik. Springer-Lehrbuch: 2016. pp. 29–103.

[70] Hoekstra R, Vugteveen J, Warrens MJ, Kruyen PM. An empirical analysis of alleged misunderstandings of coefficient alpha. Int J Soc Res Methodol. 2018;22(4):351–64.

[71] Moosbrugger H, Kelava A. Qualitätsanforderungen an Tests und Fragebogen („Gütekriterien"). In: Moosbrugger H, Kelava A, editors. Testtheorie und Fragebogenkonstruktion. Berlin, Heidelberg: Springer; 2020. pp. 13–38.

[72] Horvat J. Questionnaire. In: Lovric M, editor. International Encyclopedia of Statistical Science. Springer Berlin Heidelberg; 2011. pp. 1154–6.

[73] Sudeck G, Pfeifer K. Physical activity-related health competence as an integrative objective in exercise therapy and health sports – conception and validation of a short questionnaire. Sportwiss. 2016;46:74–87.

[74] Carl J, Sudeck G, Pfeifer K. Competencies for a Healthy Physically Active Lifestyle-Reflections on the Model of Physical Activity-Related Health Competence. J Phys Act Health. 2020;17(7):688–97. 32473589 10.1123/jpah.2019-0442

