# Supplementary material for: Focus group-supported development and psychometric exploration of an instrument to assess perceived physical exertion in nursing students
Source: BMC Nurs. 2024 Dec 30;23:957. doi: 10.1186/s12912-024-02639-9 (PMC11687016; doi:10.1186/s12912-024-02639-9)
Supplement: Supplementary file 1 — Supplementary Material 1 [file 12912_2024_2639_MOESM1_ESM.pdf]

Questions of the questionnaire that were reduced after the third focus group interview

Original (German) Item	English-translated Item
**Work-related Items**	
Sie laufen in einer Notfallsituation schnell über die Station.	You are running fast across the ward in an emergency situation.
Sie fixieren mit einer anderen Pflegekraft eine Patientin (ca. 80 kg) mit Gurten am Bett.	You and another nurse are strapping a patient (approx. 80 kg) to the bed
Sie unterstützen eine Patientin bei der Essenseinnahme.	You are helping a patient with food intake
Sie gehen ohne zusätzliche Last über die Station.	You are walking across the ward without any additional load
**School-related Items**	
Sie stehen 15 Minuten lang auf einer Stelle (z.B. in der Pause).	You are standing in one place for 15 minutes (e.g., during break)
