# Supplementary material for: Focus group-supported development and psychometric exploration of an instrument to assess perceived physical exertion in nursing students
Source: BMC Nurs. 2024 Dec 30;23:957. doi: 10.1186/s12912-024-02639-9 (PMC11687016; doi:10.1186/s12912-024-02639-9)
Supplement: Supplementary file 2 — Supplementary Material 2 [file 12912_2024_2639_MOESM2_ESM.pdf]

Questionnaire in German language

**Stellen Sie sich folgende Tätigkeiten im Pflegealltag vor:**	**Wie sehr beansprucht Sie das körperlich?**					
	gar nicht				stark	keine Angabe
Sie positionieren eine immobile Patientin (ca. 80 kg) ohne ihre Unterstützung im Bett.	O 0	O 1	O 2	O 3	O 4	O 9
Sie positionieren einen immobilen Patienten mit Adipositas (ca. 120 kg) ohne seine Unterstützung im Bett.	O 0	O 1	O 2	O 3	O 4	O 9
Sie mobilisieren eine immobile Patientin (ca. 80 kg) ohne ihre Unterstützung vom Bett in den Rollstuhl.	O 0	O 1	O 2	O 3	O 4	O 9
Sie mobilisieren einen teilmobilen Patienten (ca. 80 kg) mit seiner Unterstützung vom Bett in den Rollstuhl.	O 0	O 1	O 2	O 3	O 4	O 9
Sie mobilisieren eine teilmobile Patientin mit Adipositas (ca. 120 kg) mit ihrer Unterstützung vom Bett in den Rollstuhl.	O 0	O 1	O 2	O 3	O 4	O 9
Sie mobilisieren eine teilmobile Patientin (ca. 80 kg) nach einem Sturz mit einer zweiten Pflegekraft vom Boden ins Bett.	O 0	O 1	O 2	O 3	O 4	O 9
Sie waschen einen immobilen Patienten (ca. 80 kg) ohne seine Unterstützung im Bett.	O 0	O 1	O 2	O 3	O 4	O 9
Sie waschen eine immobile Patientin mit Adipositas (ca. 120 kg) ohne ihre Unterstützung im Bett.	O 0	O 1	O 2	O 3	O 4	O 9
Sie unterstützen einen teilmobilen Patienten (ca. 80 kg) bei der Körperpflege in einem engen Bad.	O 0	O 1	O 2	O 3	O 4	O 9
Sie entleeren nacheinander den Dauerkatheter bei 8 Patientinnen und Patienten.	O 0	O 1	O 2	O 3	O 4	O 9
Sie ziehen einer Patientin (ca. 80 kg) im Bett Kompressionsstümpfe an.	O 0	O 1	O 2	O 3	O 4	O 9
Sie reinigen die Wunde eines Patienten (ca. 80 kg) an einer schwierig zu erreichenden Stelle (z. B. Wade, Gesäß).	O 0	O 1	O 2	O 3	O 4	O 9
Sie wechseln den Verband am Bein einer Patientin (ca. 80 kg) und halten dabei das Bein hoch.	O 0	O 1	O 2	O 3	O 4	O 9
Sie messen nacheinander bei 20 Patientinnen und Patienten den Blutdruck.	O 0	O 1	O 2	O 3	O 4	O 9
Sie stecken nacheinander bei 20 Patientinnen und Patienten Infusionen an und ab.	O 0	O 1	O 2	O 3	O 4	O 9
Sie stellen die Medikamente für 20 Patientinnen und Patienten.	O 0	O 1	O 2	O 3	O 4	O 9

Sie beziehen innerhalb von 5 Minuten ein leeres Bett neu.	O0	O1	O2	O3	O4	O9
Sie desinfizieren innerhalb von 2 Minuten die Oberflächen eines Zimmers (z. B. Fernseher, Patientenruf).	O0	O1	O2	O3	O4	O9
Sie stehen 20 Minuten lang auf einer Stelle (z. B. im OP, beim Dokumentieren).	O0	O1	O2	O3	O4	O9
Sie steigen die Treppe über 2 Stockwerke hinauf.	O0	O1	O2	O3	O4	O9
Sie transportieren Gegenstände bis zu 5 kg (z. B. Tee, Handtücher, Essenstabletts) über die Station.	O0	O1	O2	O3	O4	O9
Sie transportieren Gegenstände über 5 kg (z. B. Gepäck eines Patienten) von einer Station zur nächsten.	O0	O1	O2	O3	O4	O9
Sie schieben ein leeres Bett über die Station.	O0	O1	O2	O3	O4	O9
Sie schieben einen Patienten im Rollstuhl von einer Station zur nächsten.	O0	O1	O2	O3	O4	O9
Sie heben Gegenstände bis zu 5 kg (z. B. Karton mit Medikamenten) hoch.	O0	O1	O2	O3	O4	O9
Sie heben Gegenstände über 5 kg (z. B. Spüllösungen, Sauerstoffflaschen) hoch.	O0	O1	O2	O3	O4	O9
Sie halten Gegenstände bis zu 5 kg (z. B. Infusionsbeutel) über einen Zeitraum von 1 Minute.	O0	O1	O2	O3	O4	O9
Sie halten Gegenstände über 5 kg (z. B. Spüllösungen) über einen Zeitraum von 1 Minute.	O0	O1	O2	O3	O4	O9

**Stellen Sie sich folgende Tätigkeiten im Schulalltag vor:**	**Wie sehr beansprucht Sie das körperlich?**					
	gar nicht				stark	keine Angabe
Sie sitzen während eines gewöhnlichen Schultages im Unterricht.	O0	O1	O2	O3	O4	O9
Sie tragen Ihre Schulsachen (z. B. Tasche, Ordner, Bücher/Tablet) während eines gewöhnlichen Schultages mit sich herum.	O0	O1	O2	O3	O4	O9
