# Supplementary material for: Focus group-supported development and psychometric exploration of an instrument to assess perceived physical exertion in nursing students
Source: BMC Nurs. 2024 Dec 30;23:957. doi: 10.1186/s12912-024-02639-9 (PMC11687016; doi:10.1186/s12912-024-02639-9)
Supplement: Supplementary file 3 — Supplementary Material 3 [file 12912_2024_2639_MOESM3_ESM.pdf]

Questionnaire in English translation

**Imagine the following activities in your everyday working life:**	**How much physically demanding is it for you?**					
	Not at all				Very much	N/A
You are positioning an immobile patient (approx. 80 kg) in bed without their help.	O_0	O_1	O_2	O_3	O_4	O_9
You are positioning an immobile patient with obesity (approx. 120 kg) in bed without their help.	O_0	O_1	O_2	O_3	O_4	O_9
You are moving an immobile patient (approx. 80 kg) from bed to a wheelchair without their help.	O_0	O_1	O_2	O_3	O_4	O_9
You are moving a partially mobile patient (approx. 80 kg) from bed to a wheelchair with their help.	O_0	O_1	O_2	O_3	O_4	O_9
You are moving a partially mobile patient with obesity (approx. 120 kg) from bed to a wheelchair with their help.	O_0	O_1	O_2	O_3	O_4	O_9
You are moving a partially mobile patient (approx. 80 kg) from the ground to bed together with a second nurse after a fall.	O_0	O_1	O_2	O_3	O_4	O_9
You are washing an immobile patient (approx. 80 kg) in bed without their help.	O_0	O_1	O_2	O_3	O_4	O_9
You are washing an immobile patient with obesity (approx. 120 kg) in bed without their help.	O_0	O_1	O_2	O_3	O_4	O_9
You are assisting a partially mobile patient (approx. 80 kg) in body care in a narrow bathroom.	O_0	O_1	O_2	O_3	O_4	O_9
You are emptying indwelling catheters of 8 patients one after another.	O_0	O_1	O_2	O_3	O_4	O_9
You are putting on compression stockings on a patient (approx. 80 kg) in bed.	O_0	O_1	O_2	O_3	O_4	O_9
You are cleaning the wound of a patient (approx. 80 kg) in an area that is difficult to reach (e.g., calf, buttocks).	O_0	O_1	O_2	O_3	O_4	O_9
You are changing the bandage on a leg of a patient (approx. 80 kg) while holding up the leg.	O_0	O_1	O_2	O_3	O_4	O_9
You are measuring the blood pressure of 20 patients one after another.	O_0	O_1	O_2	O_3	O_4	O_9
You are connecting and disconnecting infusions of 20 patients one after another.	O_0	O_1	O_2	O_3	O_4	O_9
You are preparing the medication for 20 patients.	O_0	O_1	O_2	O_3	O_4	O_9

You are putting clean sheets on an empty bed within 5 minutes.	O0	O1	O2	O3	O4	O9
You are disinfecting the surfaces in a room (e.g., television, nurse call button) within 2 minutes.	O0	O1	O2	O3	O4	O9
You are standing in one place for 20 minutes (e.g., during surgery, while documentation).	O0	O1	O2	O3	O4	O9
You are climbing the stairs over 2 floors.	O0	O1	O2	O3	O4	O9
You are transporting objects up to 5 kg (e.g., tea, towels, food trays) across the ward.	O0	O1	O2	O3	O4	O9
You are transporting objects above 5 kg (e.g., luggage of a patient) from one ward to the next.	O0	O1	O2	O3	O4	O9
You are pushing an empty bed across the ward.	O0	O1	O2	O3	O4	O9
You are pushing a patient in a wheelchair from one ward to the next.	O0	O1	O2	O3	O4	O9
You are lifting up objects up to 5 kg (e.g., carton with medication).	O0	O1	O2	O3	O4	O9
You are lifting up objects above 5 kg (e.g., rinsing solutions, oxygen bottles).	O0	O1	O2	O3	O4	O9
You are holding objects up to 5 kg (e.g., infusion bags) over 1 minute.	O0	O1	O2	O3	O4	O9
You are holding objects above 5 kg (e.g., rinsing solutions) over 1 minute.	O0	O1	O2	O3	O4	O9

**Imagine the following activities in your everyday school life:**	**How much physically demanding is it for you?**					
	Not at all				Very much	N/A
You are sitting in class on a usual school day.	O0	O1	O2	O3	O4	O9
You are carrying school things (e.g. bag, folder, books/tablet) with you on a usual school day.	O0	O1	O2	O3	O4	O9
