# Supplementary material for: Focus group-supported development and psychometric exploration of an instrument to assess perceived physical exertion in nursing students
Source: BMC Nurs. 2024 Dec 30;23:957. doi: 10.1186/s12912-024-02639-9 (PMC11687016; doi:10.1186/s12912-024-02639-9)

Methods to determine the factor number:

Table 1: Eigenvalue

Explained total variance

	Initial Eigenvalues			Sums of squared factor loadings for extraction		
Factor	Total	% of variance	Cumulated %	Total	% of variance	Cumulated %
1	14.217	47.390	47.390	13.908	46.360	46.360
2	3.533	11.778	59.168	3.191	10.638	56.998
3	1.333	4.442	63.609	.999	3.329	60.327
4	1.148	3.827	67.437	.805	2.682	63.009
5	1.005	3.349	70.786	.720	2.401	65.410
6	.896	2.987	73.772			
7	.824	2.748	76.520			
8	.740	2.466	78.987			
9	.624	2.079	81.065			
10	.539	1.797	82.863			
11	.517	1.723	84.586			
12	.504	1.679	86.265			
13	.434	1.448	87.713			
14	.375	1.251	88.964			
15	.357	1.189	90.154			
16	.343	1.144	91.297			
17	.323	1.078	92.375			
18	.309	1.031	93.406			
19	.280	.932	94.338			
20	.251	.836	95.174			
21	.222	.741	95.914			
22	.209	.698	96.612			
23	.191	.638	97.250			
24	.171	.571	97.820			
25	.156	.521	98.342			
26	.136	.455	98.797			
27	.128	.427	99.224			
28	.099	.331	99.555			
29	.070	.234	99.789			
30	.063	.211	100.000			

Extraction method: Principal Axis Factoring

Figure 1: Scree Plot

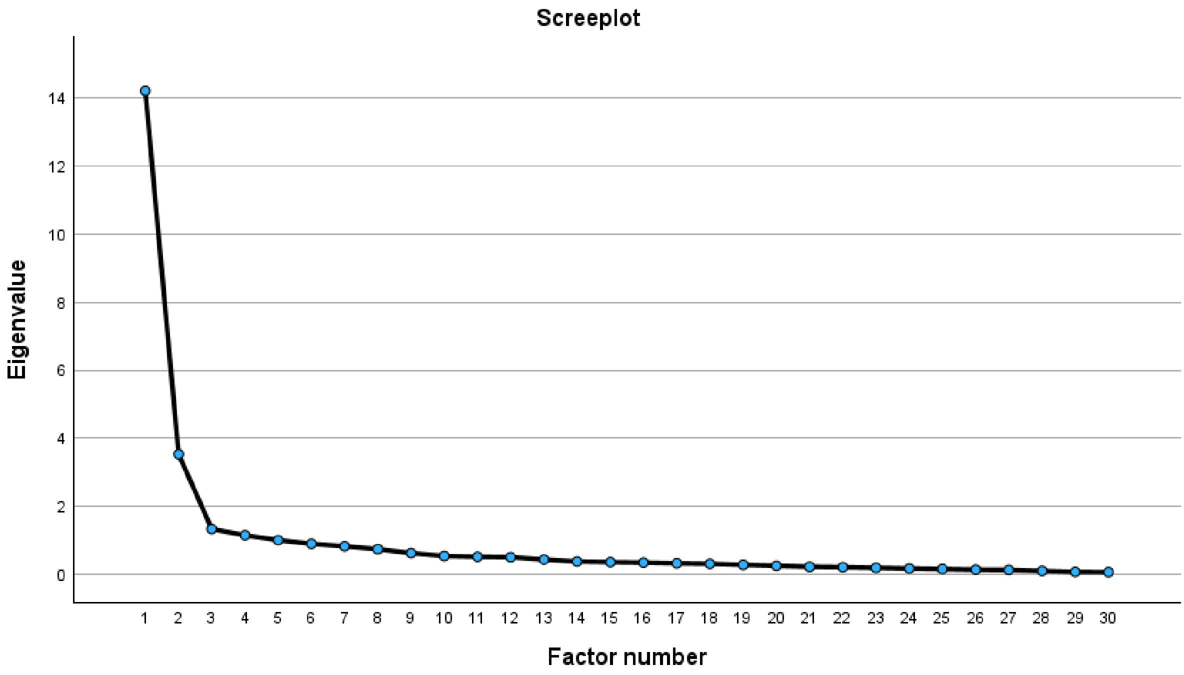

Velicier's MAP Test:

Run MATRIX procedure:

Velicer's Minimum Average Partial (MAP) Test:

Eigenvalues

14.2169
3.5334
1.3325
1.1481
1.0047
.8960
.8244
.7399
.6236
.5392
.5170
.5037
.4345
.3752
.3568
.3431
.3233
.3093
.2796
.2509
.2222
.2093
.1913
.1712
.1563
.1365
.1282
.0992
.0703
.0634

Average Partial Correlations

	squared	power4
,0000	.2219	.0808
1.0000	.0355	.0046
2.0000	.0209	.0016
3.0000	.0186	.0012
4.0000	.0186	.0011
5.0000	.0209	.0020
6.0000	.0226	.0018
7.0000	.0235	.0020
8.0000	.0251	.0023
9.0000	.0270	.0035
10.0000	.0302	.0040

11.0000	.0327	.0061
12.0000	.0353	.0062
13.0000	.0398	.0076
14.0000	.0458	.0102
15.0000	.0519	.0125
16.0000	.0598	.0143
17.0000	.0701	.0177
18.0000	.0802	.0205
19.0000	.0883	.0266
20.0000	.1085	.0385
21.0000	.1280	.0484
22.0000	.1506	.0613
23.0000	.1790	.0711
24.0000	.1852	.0771
25.0000	.2126	.1041
26.0000	.2665	.1402
27.0000	.3153	.1855
28.0000	.4843	.3584
29.0000	1.0000	1.0000

The smallest average squared partial correlation is
.0186

The smallest average 4rth power partial correlation is
.0011

The Number of Components According to the Original (1976) MAP Test is
3

The Number of Components According to the Revised (2000) MAP Test is
4

Figure 2: Parallel analysis PAA

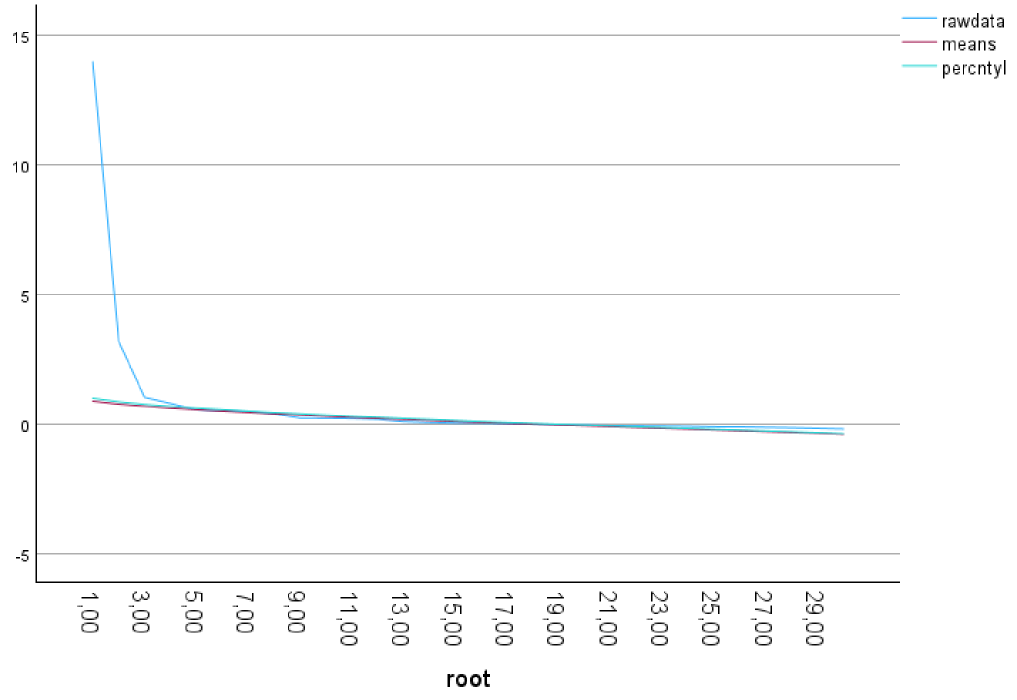

Figure 3: Parallel analysis PCA

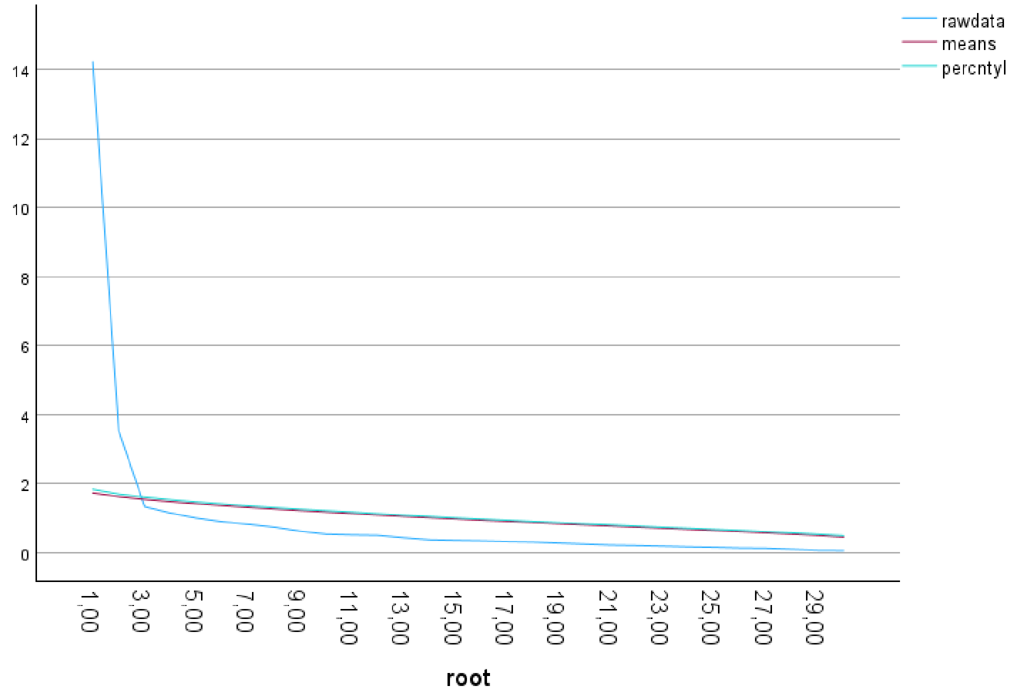

Table 2: Chi Quadrat (ML-Test):

**Factors**	**df**	**Chi^2**	**p**
2	376	1430.76	<.001
3	348	1137.98	<.001
4	321	926.34	<.001
5	295	803.71	<.001
6	270	705.03	<.001
7	246	589.98	<.001
8	223	491.72	<.001
9	201	400.52	<.001
10	180	320.99	<.001
11	160	260.68	<.001
12	141	210.12	<.001
13	123	164,.3	.007
14	106	136.14	.026
15	90	95.3	.331

From factor 4 on with Promax rotation

Figure 4: EKC

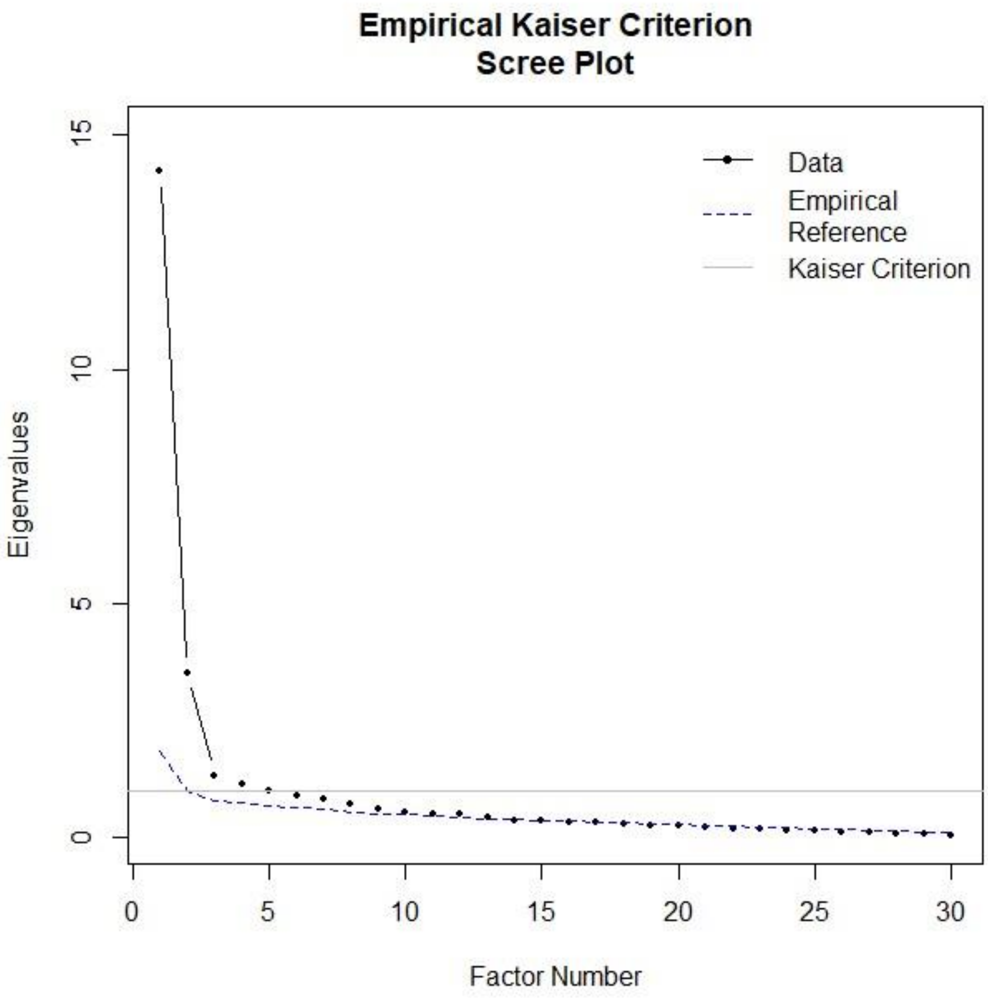

Supplement: Supplementary file 4 — Supplementary Material 4 [file 12912_2024_2639_MOESM4_ESM.pdf]
