# Supplementary material for: Focus group-supported development and psychometric exploration of an instrument to assess perceived physical exertion in nursing students
Source: BMC Nurs. 2024 Dec 30;23:957. doi: 10.1186/s12912-024-02639-9 (PMC11687016; doi:10.1186/s12912-024-02639-9)
Supplement: Supplementary file 5 — Supplementary Material 5 [file 12912_2024_2639_MOESM5_ESM.pdf]

Table 1: Factor correlation matrix

Factor	1	2	3
1	1,000	.694	.094
2	.694	1.000	.262
3	.094	.262	1.000

Extraction method: Principal Axis Factoring
Rotation method: Promax with Kaiser-normalization
